# From retrieval to recovery: a joint expert perspective on equity and trauma systems reform in regional Northern Queensland, Australia

**DOI:** 10.3389/frhs.2026.1830470

**Published:** 2026-07-16

**Authors:** Breeanna Spring, Anna Grant, Vinay Gangathimmaiah, Brittney Finlayson, Benjamin J. Crowley, Clinton Gibbs, Tristan Pillay, Margot Bosanquet, Tegan Ely, Richard Franklin, Matan Ben David

**Affiliations:** 1 Townsville Hospital and Health Service, Townsville, QLD, Australia; 2 Molly Wardaguga Institute for First Nations Birth Rights, Faculty of Health, Charles Darwin University, Darwin, NT, Australia; 3 School of Medicine, Faculty of Health, Charles Darwin University, Darwin, NT, Australia; 4 James Cook University, College of Medicine and Dentistry, Townsville, QLD, Australia; 5 Queensland Digital Health Centre, The University of Queensland, Herston, QLD, Australia; 6 School of Public Health and Preventive Medicine, Monash University, Melbourne, VIC, Australia; 7 Retrieval Services Queensland, Townsville, QLD, Australia; 8 Queensland Cerebral Palsy and Rehabilitation Research Centre, The University of Queensland, Brisbane, QLD, Australia; 9 Macquarie University, School of Psychological Sciences, Sydney, NSW, Australia

**Keywords:** Aboriginal and Torres Strait Islander People, emergency medical services, program implementation, rural population, trauma centers, tropical climate

## Abstract

Tropical Northern Queensland is a geographically vast jurisdiction, in which Townsville University Hospital serves as the regional comprehensive major trauma centre. Northern Queensland trauma has been an under-examined field, with the evidence base required to characterise its burden and inform equitable care only recently beginning to emerge. In Australia, Aboriginal and Torres Strait Islander Peoples, children, older adults, and those living in regional, rural, and remote communities experience disproportionately higher rates of injury, hospitalisation, higher burden and severity of consequent impairments, and injury-related death – compounded by Northern Queensland’s demographic profile and structural inequities that drive injury risk and limit access to care across its expansive catchment. This perspective highlights the growing recognition among specialist clinicians and researchers that advancing trauma research and system development is critical for sustainable and equitable trauma care. In this perspective we introduce two early initiatives, the Townsville University Hospital Trauma Research Program and Trauma Connect – aiming to build the evidence base and foundational structures required to drive regional trauma care reform in Northern Queensland, and more broadly, Australia.

## 1 Introduction

Trauma and injures are a major contributor to morbidity and mortality in Australia, remaining one of the leading reasons for emergency department presentations and hospital admissions (1). Trauma and injuries are the leading cause of death among people aged 1–44 years, and the rates of hospitalised injury and injury related mortality increases with remoteness (1, 2). Aboriginal and Torres Strait Islander Peoples experience a disproportionately higher trauma and injury burden compared to other Australian communities (1). Recent data shows that the disparity in emergency presentations, hospitalisations and injury-related deaths between Aboriginal and Torres Strait Islander Peoples and non-Indigenous Australians continues to widen, with Aboriginal and Torres Strait Islander People experiencing injury rates more than twice those of non-Indigenous Australians (1).

### 1.1 Significance of major trauma

Major trauma refers to one or more injuries that pose an immediate threat to life and may lead to lasting disability (3). In Australia, major trauma is typically defined as an Injury Severity Score greater than 12, whereas internationally, a score of 15 is more commonly used (4). Although major trauma represents a relatively small proportion of all trauma presentations, it carries the highest risk of morbidity, mortality and resource utilisation (1, 5).

Major trauma patients nationally spend a median of 7.10 (3.64–15.00) days in hospital, and 4 (2.00–9.00) days in intensive care, reflecting the substantial clinical burden (5). As major trauma requires structured processes for the delivery of timely, efficient care, outcomes for major trauma are widely regarded as indicators of overall system performance. These structures, pathways and clinical capabilities needed to care for the severely injured patients strengthen trauma care across all acuity levels (5, 6).

### 1.2 Northern Queensland context

Northern Queensland covers one of the largest and most geographically dispersed major trauma catchments in Australia, including North and Far North Queensland (7). The Far North Queensland region includes from the Cassowary Coast far north to the Torres Strait Islands. South, the North Queensland region encompasses the Burdekin, Charters Towers, Hunchinbrook, Palm Island and Townsville (7). Responding to major trauma in the Northern Queensland region covers approximately 800,000 square kilometers, involving over 763,000 people from many isolated communities and regional areas (7–9). This geography uniquely shapes all aspects of trauma care, influencing retrieval travel times, retrieval requirements and access to definitive hospital care.

Townsville University Hospital serves as the primary tertiary referral centre for Northern Queensland, receiving major trauma from across the region’s extensive footprint. Cairns Hospital, Mackay Base Hospital and Mount Isa Base Hospital – provide public emergency and major trauma care within their local catchments, though onward transfer or aeromedical bypass directly to Townsville occurs, in particular for neurosurgical, cardiothoracic and paediatric tertiary care (9). Because of the dispersed population and long distances between facilities, in Northern Queensland there is a heavy reliance on aeromedical retrieval services, supporting regional, rural and remote healthcare facilities (2, 10). Retrieval Services Queensland, a branch of the Queensland Ambulance Service Division of the Department of Health, provides clinical and operational coordination and governance of the state’s aeromedical retrieval network. This is supported by comprehensive telehealth infrastructure, enabling early communication between rural and remote clinicians and tertiary based critical care specialists, following activation of early notification pathways for major trauma, ensuring early clinical support, timely mobilization of retrieval assets and clinical teams (11).

### 1.3 Northern Queensland unique regional health service characteristics

Northern Queensland is a tropical region of northern Australia, broadly defined by its position as above or intersecting with the tropic of Capricorn (12, 13). Townsville University Hospital is the region’s designated major trauma centre, managing around 300 major trauma cases annually—the highest volume of any regional trauma service nationally and comparable to the bi-national median (14, 15).

The catchment presents a distinct combination of factors that shape trauma demand and service delivery, including geographical isolation, a tropical climate, vast distances between communities and variable access to specialist clinical services (16). Patterns of risk taking behaviour are not yet fully defined, however preliminary audit findings indicate at least 31.4% of major trauma patients admitted to Townsville University Hospital are under the influence of alcohol and/or drugs, likely under-representing the true burden (17). These conditions collectively create a complex trauma operating environment across the region and contribute to the volume, severity and nature of injuries encountered across the region.

Predominant injury mechanisms reflect the regional context and include motor vehicle and motorcycle crashes, agricultural and occupational injuries, assaults and falls, with alcohol and substance use frequently contributing to presentations (2, 10, 17, 18). Injuries and associated hospitalisations are over represented by males, rural and remote peopleand among Aboriginal and Torres Strait Islander Peoples (10, 18–20).

Given the region’s geography, prehospital and retrieval care is central to achieving equitable trauma care and outcomes. Fixed wing, rotary wing and road retrieval services form the primary pathways to definitive care across extensive distances (21). These realities mean the “golden hour” of trauma care is a distant theory rather than reality, highlighting the importance of coordinated retrieval systems, early activation pathways and robust interfacility communication.

## 2 Establishment and implementation of the townsville trauma research program and trauma connect project

### 2.1 Trauma quality registries informing contextualised regional research

In 2022, Dobson, Gibbs (16) identified an urgent need for accurate data from all trauma service providers across Australia. They raised the specific concern that centralization of the data within the Australian Trauma Registry around metropolitan centres risks masking the inequities experienced in rural and remote trauma care, and highlighted the paucity of regional trauma data analysis in Northern Queensland (16).es in outcomes for regional and remote communities, there is still limited research

Progress has been made in registry capacity at Townsville University Hospital, with the Townsville Trauma Registry instituted in 2020, following 2012 closure of the state-based Queensland trauma registry (inclusive of Townsville Hospital) as a result of insufficient funding (22, 23). This contemporary regional trauma registry captures trauma admissions to the major trauma centre, with data collection aligned to the Australian New Zealand Bi-national Trauma minimum dataset, enabling prehospital and hospital-level data collection on processes and outcomes to facilitate benchmarking, quality improvement and research, with submissions included in the Australian New Zealand Trauma Registry since 2020 (14). Establishing this registry has been foundational to the development of the Trauma Research Program.

Elsewhere within the catchment, trauma registries are established at Mackay Base Hospital and Mt Isa Base Hospital - regional and remote sites respectively - though with limited associated research output, and without current capacity for submission to the Bi-national registry for benchmarking.

The establishment of the Queensland Trauma Data Collection in 2021 represents a significant advance for statewide trauma system performance monitoring and evaluation, research, and clinical quality initiatives across Queensland, including rural and remote sites (24). This initiative has provided statewide visibility of trauma outside major trauma centres, with almost half of cases managed entirely at regional or rural sites (24).

New epidemiological evidence by Miyata, Asomah (18) demonstrates the potential of registry capacity to enable research, identifying a significant trauma burden in remote North-West Queensland with unique demographic and injury profiles amongst priority populations. System limitations are highlighted, with concerns regarding data quality that impairs analysis and reporting on Aboriginal and Torres Strait Islander Peoples - important considerations for research across regional and remote health services.

### 2.2 Establishing the townsville trauma research program

In April 2024, a multidisciplinary group of clinicians and researchers at Townsville University Hospital, in collaboration with representatives from Retrieval Services Queensland and partner retrieval organisations, established the Trauma Research Program to strengthen trauma research capacity and provide a coordinated platform for system level improvement. The Trauma Research Program was conceived to generate and implement high quality evidence, support clinical innovation and enhance contextualised trauma care across Northern Queensland’s geographically dispersed catchment.

The Trauma Research Program reflects a shared commitment to improving outcomes for communities that experience significant geographic, cultural and service delivery challenges. The Trauma Research Program reflects a shared commitment to improving outcomes for communities across Northern Queensland, where rural and remote contexts continue to experience significantly poorer health outcomes. These disparities are driven by the realities of distance, limited-service access, workforce constraints, and systems that have not consistently met the cultural and contextual needs of communities. Together, these factors compound the impacts of trauma and may delay timely, effective care. This work is grounded in addressing both the outcomes and the underlying conditions that continue to drive them.

The Townsville Trauma Research Program is anchored by a centralized governance structure overseeing five strategic pillars – injury prevention, improved trauma care, trauma systems development, new models and pathways, and long-term outcomes – with an emphasis on reducing preventable mortality, geographical health disparities and improving functional recovery. Underpinning all research domains is a foundational commitment to culturally responsive, community-centered care for Aboriginal and Torres Strait Islander Peoples and vulnerable populations across Northern Queensland, including advancing care closer to home.

### 2.3 Building research and outreach capacity

The Townsville Trauma Research Program received two years of funding from the Townsville Hospital and Health Service Study, Education and Research Trust Account to establish dedicated Clinical Research Coordinator capacity. This investment enabled the development and delivery of trauma research activities, including coordination, governance, data quality assurance, and knowledge translation. Establishing this foundational research infrastructure created the capability required to support sustained trauma research activity and embed clinical engagement within a busy regional tertiary centre.

Additional funding supported the launch of the Motor Accident Insurance Commission funded *Trauma Connect project*, one of the key early initiatives and important component in strengthening the region’s trauma research platform. The Trauma Connect project was organically conceived by expert clinicians recognising that trauma care in Northern Queensland is complex, required maturation and a networked approach to systems development and research initiatives. The Trauma Connect project embeds clinician researchers within the trauma service, strengthening trauma network collaboration, trauma specialist care co-ordination and continuity, capacity building, and research translation. The project scope includes Trauma Connect clinics, trauma outcomes follow-up, co-ordination of trauma care, trauma research development, trauma navigation, and trauma governance and system development.

The program extends expert trauma care co-ordination and system evaluation by providing a consistent model of follow-up beyond hospital discharge through structured follow-up processes, navigation and outcome measurement after discharge from the major trauma centre, addressing longstanding gaps in recovery, care continuity, and person-centred outcomes (25). The program also serves as an outreach mechanism, strengthening communication pathways with regional, rural and remote care providers and improving visibility of post-discharge needs and service gaps across the trauma continuum. These developments have improved the trauma system to be better connected, responsive, and regionally attuned to the communities it serves.

Trauma Connect program evaluation will focus on feasibility of follow-up services and outcome evaluation, feasibility of data collection, and feasibility of clinical-research hybrid roles in contributions to trauma clinical and quality activities, capacity building activities, trauma system development activities, and research activities (grants, active projects, dissemination).

Further capacity building has been supported through the Emergency Medicine Foundation grant, which has enabled the expansion of trauma research activity across Northern Queensland (26). This initiative is supporting capacity building – clinician led research, whilst enhancing the translation of evidence into practice across diverse and dispersed settings. This investment has contributed to broadening the program’s reach and reinforced the role of the Townsville Trauma Research Program driving trauma system improvement across the Northern Queensland region.

### 2.4 Barriers

Implementation has occurred within a complex clinical and geographic environment, and several barriers have been identified. Clinicians face significant workload pressures, limiting the availability of protected time for research engagement and project leadership. The vast geographic dispersion of the trauma and injury catchment complicates consistent follow-up, stakeholder engagement and equitable access to post-discharge services and research opportunities, particularly for patients and clinicians in remote communities. Variability in research literacy and experience across disciplines and regional sites further highlights the need for ongoing capacity building and tailored support and contributes to challenges for multi-site initiatives. These challenges underscore the importance of sustained investment, collaborative partnerships and flexible models of engagement, to ensure trauma research and outreach efforts remain feasible, impactful and sustainable across North Queensland.

The first phase of this research program includes a range of small and large projects, such as Trauma Connect, to explore and describe audit current service delivery. These audit initiatives help to describe the service provision within the hospital and have identified key issues including workforce, capacity in research, system resource constraint and unique population profile. The next phase will use implementation science to develop a program of work to help implement high quality evidence, support clinical innovation and enhance contextualised trauma care (27).

### 2.5 Enablers

Despite these challenges, several enablers have supported early progress. Strong clinical leadership across trauma, emergency, surgical, intensive care, retrieval, allied health, rehabilitation services and Indigenous health services has fostered shared ownership of the Trauma Research Program’s objectives. The establishment of dedicated research coordination has improved study feasibility, governance compliance and data quality. Trauma Connect has strengthened a culture of clinical research and innovation across the trauma team. A collaborative culture between Townsville University Hospital, regional hospitals, retrieval services and community-based providers has expanded outreach capacity and created new opportunities for system-integrated research. This is the first phase focused on situational analysis, building research capacity and establishing collaborative partnerships to identify and describe critical gaps in trauma care and research priorities. The next phase will draw on implementation science to optimise translational and targeted research designs. Although the program was not prospectively designed using an implementation framework, these foundational enabling conditions have proven crucial to the early progress and are essential for program uptake and sustainability in any future scale-up (28).

Institutional support has positioned the Trauma Research Program as both strategically aligned and inherently innovative, grounded in an understanding that trauma outcomes in North Queensland are shaped by factors unique to First Nations peoples and the realities of geographical remoteness. By recognising how culture, Country, distance, and service access intersect to influence recovery, the program enables new and responsive research pathways that improve outcomes for Aboriginal and Torres Strait Islander peoples, in line with THHS’s Health Equity Strategy and national Closing the Gap priorities. This approach is strengthened through a commitment to culturally inclusive research practice, building sustainable clinician & researcher pathways, and fostering cross-disciplinary collaboration. Together, it aims to advance models of care that bring treatment closer to home while enhancing patient outcomes and experience, reinforcing the program’s strategic value and system-wide impact.

### 2.6 Governance

The Trauma Research Program operates within Townsville University Hospital’s established research governance structures, ensuring ethical oversight, data stewardship, and alignment with organisational strategy. Trauma Connect is embedded within clinical governance pathways, enabling coordinated communication between acute care, rehabilitation, primary care, and community services. Together, these structures support consistent standards, shared accountability, and system wide visibility of trauma processes and outcomes.

### 2.7 Workforce and system-level considerations

Sustaining trauma research and outreach across geographically dispersed regions requires deliberate attention to workforce and system-level factors, particularly the strength and stability of the First Nations workforce across both clinical and non-clinical roles. In rural and remote settings, Aboriginal and Torres Strait Islander staff are central to continuity of care, cultural safety, and engagement, with their presence strongly influencing recovery outcomes. Ensuring robust Aboriginal and Torres Strait Islander workforce representation is not only a matter of equity, but a determinant of clinical effectiveness.

Clinical services across Northern Queensland operate under substantial workload pressures, particularly in key specialties, and high turnover in rural and remote facilities, and limited protected time for research, quality improvement and follow-up activities continue to challenge both service delivery and long-term sustainability of trauma initiatives. These pressures compromise the continuity of care and impede on the establishment of enduring research partnerships across the region. System level coordination is further shaped by the distributed nature of the trauma network, necessitating consistent communication pathways, shared protocols, and reliable mechanisms for integrating research activity into routine clinical practice.

Strengthening partnerships with local First Nations workforce, partnering Hospital and Health Services, and community-controlled health services is therefore critical to addressing these challenges. This work is supported through the Townsville Hospital and Health Service Aboriginal and Torres Strait Islander Workforce Strategy within the broader Health Equity framework. Together, these considerations underscore the need for coordinated workforce planning, sustained investment in research capability, and ongoing support for regional and community partners to ensure meaningful and long-term impact.

### 2.8 Early outcomes and outputs

Despite these challenges, the Trauma Research Program and Trauma Connect project have generated several early outcomes that demonstrate their value to the Northern Queensland trauma system. Improvements in trauma data completeness and accuracy have strengthened the region’s capacity to generate high-quality research describing injury patterns, identifying service gaps, and informing targeted quality improvement initiatives. Clinician-led research activity has increased substantially, supported by dedicated coordination, strengthened governance processes, and focused capacity building initiatives.

Trauma Connect has complemented existing programs and resources by improving post-discharge engagement with specialist trauma clinicians. This has enabled more streamlined follow-up, enhanced navigation and care coordination, improved communication pathways, and reduced the risk of fragmentation across the trauma continuum. Earlier identification of unmet needs, improved data collection, and strengthened continuity of care have also contributed to consistent recovery pathways for patients and their families, regardless of geography.

Collectively these early outputs illustrate the potential of coordinated research infrastructure and outreach initiatives to enhance both patient-level outcomes and system-level understanding of trauma care in Northern Queensland.

### 2.9 System-level relevance

The Trauma Research Program and Trauma Connect project hold significant system-level relevance for trauma care across Northern Queensland and, more broadly, for regional trauma systems in Australia. By strengthening research infrastructure and enhancing communication across the trauma continuum, these initiatives are building a regional evidence base that contributes to the broader body of trauma literature – addressing a gap that has long limited the capacity for equity-driven, contextually informed decision-making in regional Australian trauma care. The insights generated through the Trauma Research Program support evidence-informed resource allocation and highlight areas where targeted investment may improve equity and outcomes for priority populations. Trauma Connect project’s outreach function reinforces the importance of continuity of care across vast distances and diverse communities utilising integrated technology such as virtual clinics and telehealth, demonstrating how structured follow-up and longitudinal care coordination can mitigate geographic disadvantage. Collectively, these initiatives offer a replicable model for coordinated research and outreach in regions characterised by remoteness, population diversity, and complex service delivery environments.

## 3 Actionable recommendations

### 3.1 Lessons learnt

Early implementation of the Trauma Research Program and Trauma Connect project has demonstrated that establishing research infrastructure alongside post-discharge follow-up—rather than treating them as separate, sequential steps— is both practical and synergistic in regional trauma care. Dedicated research coordination has proven essential for maintaining data quality, supporting ethics and governance processes, and enabling clinicians to participate meaningfully in research despite competing clinical demands and varying levels of research literacy.

This first phase, comprising situational analyses and clinical audits, is generating valuable and novel insight into patient characteristics and trauma service provision in a unique tertiary setting outside a metropolitan context. For the first time, system-level visibility is identifying data gaps for targeted resourcing to drive equity-informed improvement across a geographically dispersed and culturally diverse population. The Trauma Research Program’s experience to date highlights the need for flexible models of engagement with regional, rural and remote partners, recognising that sustained collaboration requires tailored communication strategies, shared platforms for study design, and ongoing relationship building. The lessons emerging from this phase – including the realities of workforce capacity, research infrastructure, and the complexities of serving remote and First Nations communities – are themselves a contribution, and provide a critical foundation for the next phase.

The next phase will embed implementation science frameworks to ensure these lessons directly inform program improvement. Addressing the contextual and structural factors unique to this setting – including regional healthcare costs, specialist access and system-level processes – requires implementation of frameworks from the outset. It is proposed that the RE-AIM (Reach, Effectiveness, Adoption, Implementation, Maintenance) would be applied to strengthen the evaluation of the future program phases, ensuring early lessons feed into a rigorous process of improvement (29)

### 3.2 Equity considerations

Equity has emerged as a central and non-negotiable theme across both the Trauma Research Program and Trauma Connect project. The vast geography of North Queensland, combined with the disproportionate burden of injury among Aboriginal and Torres Strait Islander Peoples and residents of remote communities, underscores the need for trauma initiatives that explicitly address structural and cultural barriers to equitable care (10). Trauma Connect’s outreach function has begun to mitigate some of these inequities by improving continuity of care and strengthening communication between Townsville University Hospital and regional clinicians. Early implementation has reinforced that equitable trauma care requires system-level strategies, including culturally safe models of follow-up, improved access to rehabilitation services, and mechanisms to ensure remote communities are not disadvantaged by distance or workforce shortages. Embedding equity considerations into research priorities, data collection and program design remains essential for ensuring that trauma system improvements benefit all populations across the region, and both described programs are culturally inclusive, with focus on vulnerable populations for this reason.

Equity requires operational implementation, not merely acknowledgement (30). Two mechanisms are particularly relevant within the Trauma Research Program. First, structured post-discharge follow-up via telehealth directly addresses the geographic access barrier that disproportionately affects patients in remote communities. Second, investment in registry data quality addresses a form of epistemic inequity, populations in this regional catchment with the highest injury burden are often the least visible in trauma and injury evidence bases (31, 32). Critically, the early embedding of the Indigenous Health Service within both programs – involved across processes and decision points from the outset – moves equity from principle to practice, grounding these initiatives in the cultural safety infrastructure that the equitable implementation literature identifies as essential (33).

### 3.3 Workforce and governance

Workforce capacity and governance structures have been critical determinants of early progress, and both require sustained investment to ensure long-term viability. Protected time for clinicians to engage in research, quality improvement and outreach activities is essential, particularly in high demand environments where clinical workload routinely limits participation. Addressing workforce shortages in key specialties, especially across rural and remote areas, requires system-level planning that recognizes research capacity as integral to, rather than separate from, sustainable trauma care. Governance structures at Townsville University Hospital have provided a strong foundation for the Townsville Trauma Research Program, ensuring ethical oversight, data stewardship, and alignment with organisational priorities. The integration of Trauma Connect project into existing clinical governance pathways has strengthened communication across the trauma continuum and reinforced shared accountability for patient outcomes. Together, these considerations point to the importance of long-term investment in capability, coordination, and system leadership as preconditions for equitable, evidence-driven regional trauma care.

### 3.4 Closing statement

North Queensland’s unique cultural, geographical and demographic complexity presents both an imperative and an opportunity for trauma implementation research. The early experience of the Trauma Research Program and Trauma Connect project demonstrates that even modest, targeted investment in research coordination, post-discharge follow-up and regional partnership can generate meaningful improvements in system visibility, data quality and continuity of care — particularly for populations historically underserved by both trauma services and the research that should inform them.

Establishing dedicated research coordination is a foundational step that enables clinicians to participate in research without compromising clinical care. Structured post-discharge follow-up, particularly when delivered through an outreach-oriented model, can substantially improve continuity of care in regions characterised by distance and service fragmentation. Meaningful engagement with regional and remote partners requires deliberate strategies that recognise local contexts, workforce constraints and the importance of culturally safe care. These insights highlight the potential for coordinated research and outreach to strengthen trauma systems in geographically dispersed regions and provide a model for similar jurisdictions across Australia.

## Data Availability

Publicly available datasets were analyzed in this study. This data can be found here: not applicable, perspective piece.
